# Effects of Multiple Factors on the Compressive Strength of Porous Ceramsite Prepared from Secondary Aluminum Dross

**DOI:** 10.3390/ma17235774

**Published:** 2024-11-25

**Authors:** Yiou Wang, Xinghan Zhu, Jinliang Zhou, Jinzhong Yang, Lu Tian, Yufei Yang

**Affiliations:** 1 Faculty of Engineering, China University of Geosciences, Wuhan 430074, China; 2 State Key Laboratory of Environmental Benchmarks and Risk Assessment, Chinese Research Academy of Environmental Sciences, Beijing 100012, China; 3 Yuyuan Ningbo Environmental Technology Co., Ltd., Ningbo 315612, China

**Keywords:** secondary aluminum dross, compressive strength, porous ceramsite, factors

## Abstract

Aluminum is one of the most in-demand nonferrous metals in the world. The secondary aluminum dross (SAD) produced during aluminum smelting is a type of solid waste that urgently requires disposal. SAD, municipal solid waste incineration fly ash, and bottom slag were used as raw materials to prepare porous ceramsite in a laboratory in this study. Multi-factor design experiments were then used to explore the influence of the sintering condition on the compressive strength to provide a basis for ceramsite preparation using SAD. The results showed that, within a certain variation range, the levels of each factor showed overall positive correlations with the ceramsite compressive strength. The contributions of the ceramsite particle size, the silicon–aluminum ratio (Si/Al), the sintering temperature, and the sintering time to the compressive strength of the porous ceramsite then decreased. The factors had a synergistic effect. The interactive effect of multiple factors on the porous ceramsite compressive strength rose with an increase in the particle size and Si/Al ratio. The average compressive strength of the porous ceramsite prepared in this study was 4.06 ± 3.71 MPa, and the maximum compressive strength was 14.13 MPa. The highest ceramsite compressive strength was achieved under a sintering temperature of 1270 °C, a particle size of 2 cm, a sintering time of 30 min, and a silicon–aluminum ratio of 1.5. In addition, there was a reaction relationship between the multiple factors involved in the sintering of the SAD-based porous ceramsite. Pilot or industrial tests should be conducted in the future based on these experiments and the intended ceramsite use.

## 1. Introduction

Industrial solid waste production has significantly increased with industrial development, and this has had a negative environmental impact globally [1]. Industrial solid waste resource utilization and harm reduction are important methods to reduce environmental pollution. Aluminum is a widely used metal [2]. A large amount of aluminum ash is produced during its smelting and production processes, which is harmful to the environment [3]. Aluminum dross is divided into primary aluminum dross (PAD) and secondary aluminum dross (SAD) [4,5]. PAD can be disposed of through heat recovery technology. However, there is no effective method for SAD treatment, and SAD potentially originates from PAD and other smelting processes. Thus, achieving safe and effective SAD treatment remains an important problem [6,7].

SAD is generated by the secondary processing of PAD or aluminum waste smelting [8]. Each ton of refined aluminum typically produces 8%–15% of SAD [9]. Currently, more than 90% of SAD is directly dumped or landfilled because it is difficult to use SAD for other processes [10]. However, SAD contains pollutants, such as aluminum nitride (AlN), and it can release ammonia when in contact with water, causing air, water, and soil pollution. Therefore, SAD is not conducive to environmental protection and human health. In addition, the direct landfilling of SAD will also lead to a loss of valuable components, such as Al, AlN, and SiO_2_, which is not beneficial to resource recycling. Therefore, research on the utilization of all SAD components is necessary for sustainable development [11].

A wet process and a pyrometallurgical process are typically used for sustainable SAD utilization [11]. The wet pressure leaching process consists of the use of sodium hydroxide, hydrochloric acid, sulfuric acid, or nitric acid media to recover the primary SAD components. The metallic Al and soluble Al_2_O_3_ present in the SAD are dissolved in the aqueous solution during the chemical reaction of the leaching process. Products such as Al_2_O_3_ and AlCl_3_ can be prepared after a series of operations, such as solid–liquid separation [12,13,14,15,16]. However, the entire wet process produces harmful gasses (ammonia) that cause great harm to the surrounding environment. SAD is mixed with different additives and then sintered in a high-temperature environment for the pyrotechnic process, and this can effectively recover Al or enable the utilization of active ingredients [7]. SAD is primarily composed of Al_2_O_3_ and SiO_2_, magnesia–aluminum spinel, refractory bricks, and porcelain refractory insulation materials that can be prepared under different test environments, with significant economic benefits. In addition, SAD can be used together with other solid waste to prepare porous materials [7]. Specifically, the conversion of the AlN present in the SAD into Al_2_O_3_ and N_2_, as well as the conversion of AlC_4_ into Al_2_O_3_ and CO_2_ during the calcination process, reduces the harmfulness of SAD. The porous properties are also enhanced under high-temperature conditions. Thus, this process has good resource-based prospects for SAD [17,18].

The basic characteristics of SAD, such as high-temperature gas production and high Al content, allow SAD to be used as a raw material to smelt porous ceramsite [19,20]. Municipal solid waste incineration fly ash with high CaO content and the bottom ash with high SiO_2_ content are also included in the process to fire and mold samples. However, under the influence of multiple factors, such as the temperature, particle size, and silicon–aluminum ratio (Si/Al), the change law of the mechanical properties (such as compressive strength) of the porous ceramsite remains unclear. This limits the promotion of SAD’s utilization.

Therefore, the use of SAD to prepare ceramsite is of great significance to the circular economy and environmental protection. The aim of this study is to reveal the factors and laws that affect SAD-based ceramsite and to provide a basis for SAD resource utilization. A single-factor experiment is used to analyze the influence of the sintering conditions (e.g., sintering temperature, time, particle size, and raw material ratio) on the compressive strength of ceramsite. Orthogonal experiments are then implemented to analyze whether there is a synergistic relationship between the different influencing factors. In addition, the optimal process under multi-factor conditions for the production of porous ceramsite with good compressive strength is also studied.

## 2. Materials and Methods

**Figure 1:**
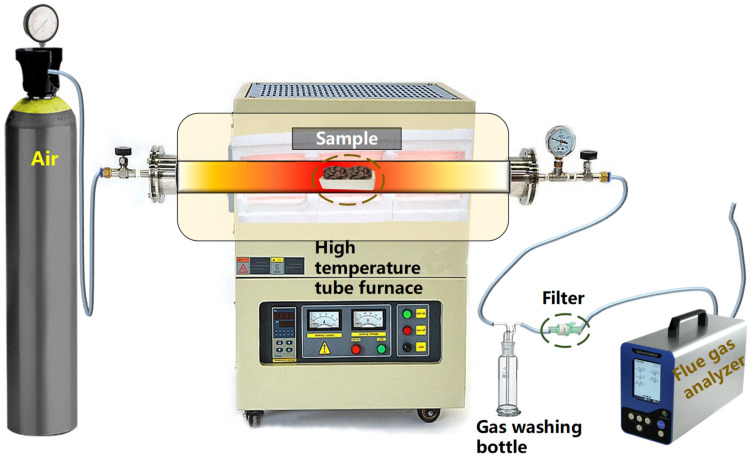
Experimental device for the sintering of porous ceramsite.

A high-temperature (1600 °C) tube furnace produced by Zhengzhou Kejia Electric Furnace Co., Ltd. was used as the sintering device (model KJ-T1600-L8010WQ, from city of LuoYang, Henan Province, China) (Figure 1). A corundum tube with a length of 1100 mm and a diameter of 80 mm was used as the heating carrier. The corundum crucible (porcelain boat), with measurements of 100 mm × 40 mm × 20 mm, was used as the sample carrier. The SAD was obtained from an aluminum ash utilization and disposal enterprise in Ningbo, China. The fly ash and bottom slag were obtained from a municipal solid waste incineration enterprise in Ningbo, China. A preliminary experiment was used to select synthetic air (composition of 21% oxygen and 79% nitrogen) to build the porous ceramsite sintering system, with a ventilation rate of 1.5 L/min. After the porous ceramsite was sintered, the compressive strengths of three ceramsites in each group were measured using a KC-3 digital particle strength tester (Gu’an Runbao Testing Technology Co., Ltd., city of LangFang, Hebei Province, China). The average value of each group was calculated to obtain the compressive strength of each ceramsite group.

Since porous ceramsite sintering is greatly affected by the experimental conditions, we used preliminary experiments to choose the specific methods for the porous ceramsite sintering experiment. These were as follows.

### 2.1. Single-Factor Test Methods

a. Sintering temperature’s effect on porous ceramsite’s compressive strength

The Si/Al value, particle size, and sintering time were 2, 2 cm, and 20 min, respectively, according to the preliminary experimental results. The sintering temperatures were 1230, 1240, 1250, 1260, and 1270 °C.

b. Sintering time’s effect on ceramsite’s compressive strength

The Si/Al value, particle size, and sintering temperature were 2, 2 cm, and 1260 °C, respectively, according to the preliminary experimental results. The sintering times were set to 10, 20, 30, and 40 min.

c. Particle size’s effect on ceramsite’s compressive strength

The Si/Al value, sintering time, and sintering temperature were 2, 20 min, and 1260 °C, respectively, according to the preliminary experimental results. The particle sizes were set to 0.5, 1.0, 1.5, and 2.0 cm.

d. Si/Al ratio’s effect on ceramsite’s compressive strength

The particle size, sintering time, and sintering temperature were 2 cm, 20 min, and 1260 °C, respectively, according to the preliminary experimental results. The Si/Al ratio was selected to have five gradients of 1, 1.5, 2, 2.5, and 3.

### 2.2. Orthogonal Test Methods

A total of 25 groups of orthogonal experiments were conducted (Table 1) based on the results of the single-factor experiments to confirm whether there was a synergistic effect between the factors.

### 2.3. Analysis Methods

The orthogonal test data were analyzed using Origin 2021 and SPSS 18.0. The Gibbs free energy changes of AlN oxidation were studied using enthalpy (H), entropy (S), and heat capacity (HSC) chemistry.

## 3. Results and Discussion

### 3.1. Effect of Sintering Temperature on Ceramsite’s Compressive Strength

**Figure 2:**
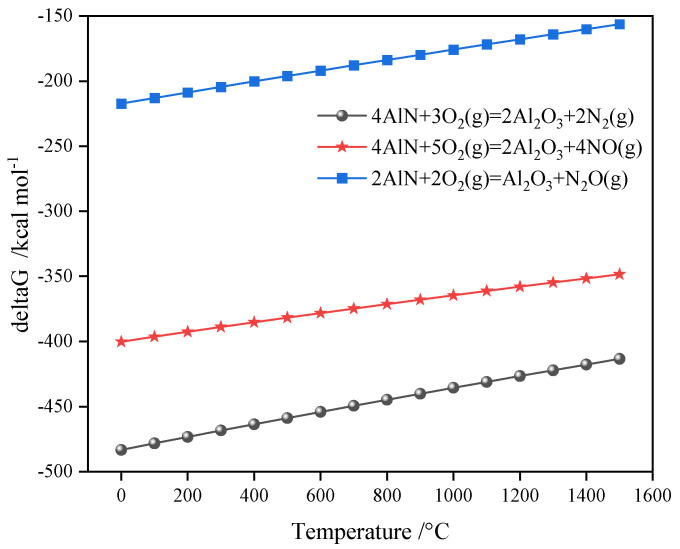
Gibbs free energy change during the AlN oxidation reaction.

The sintering temperature’s effects on the porous ceramsite’s compressive strength are shown in Table 2. The compressive strength and sintering temperature maintained an overall positive correlation. The compressive strength was the lowest at a temperature of 1250 °C. This may have been because the foaming material (AlN) in the ceramsite produced many bubbles (N_2_) (Figure 2), and this increased the number of pores inside and reduced its compressive strength [21]. The compressive strength significantly increased when the temperature was 1260 °C. This was because the foaming material in the ceramsite had essentially completed foaming, and its interior tent became stable. The compressive strength was the highest when the temperature was raised to 1270 °C. However, the molten state of the sample was due to the porous material’s collapse.

The mass loss rate in Table 2 shows that a thermal reaction occurred inside the porous ceramsite. The mass loss of the porous ceramsite first increased and then decreased within 1230–1270 °C. When the temperature reached 1250 °C, the mass loss rate was the largest. This result may have been related to volatile substances, such as water or salts, in the raw materials. The mass loss rate decrease indicated that AlN was oxidized to Al_2_O_3_. The weight increase was caused by the specific reaction process (Equations (1)–(3)) [22,23]. In [24], heating to 530–1000 °C was found to result in an increase in SAD’s weight because Al, AlN, and Al_4_C_3_ began to oxidize. The temperature of the sample in [24] was different from that found in our work. This was because the raw materials were not only aluminum ash but a mixture of aluminum ash, fly ash, and bottom slag.

The above results show that there were two types of processes occurring in the SAD-based ceramsite sintering process: (1) the release of volatile substances, such as water and salt, led to a decrease in mass, and AlN to Al_2_O_3_ oxidation led to a mass increase; and (2) AlN oxidation produced N_2_ to support the pore structure of the porous ceramsite, resulting in a decrease in compressive strength and the collapse of the ceramsite due to bubble rupture, resulting in an increase in the compressive strength. The influences of other factors, as listed below, also reflected these two reactions during the ceramsite sintering process.

4AlN + 3O_2_(g) = 2Al_2_O_3_ + 2N_2_(g); (1)

4AlN + 5O_2_(g) = 2Al_2_O_3_ + 4NO(g); (2)

2AlN + 2O_2_(g) = Al_2_O_3_ + N_2_O(g). (3)

### 3.2. Effect of Sintering Time on Ceramsite’s Compressive Strength

The porous ceramsite’s formation was greatly influenced by the sintering time. The results regarding the influence of different sintering times on the compressive strength of ceramsite are shown in Table 3. The SAD-based ceramsite’s compressive strength showed an upward trend within the sintering time of 10–20 min. This phenomenon was related to the fact that the sintering time was too long for the formation of ceramsite. The ceramsite was completely molten and collapsed when the sintering time exceeded 20 min. Thus, the compressive strength value could not be found. The ceramsite compressive strength reached the maximum value at a sintering time of 20 min. This may have been because the foaming substance (AlN) in the ceramsite was essentially foamed, and its internal solid phase structure tended to be stable [25]. In addition, the ceramsite was in a critical molten state and could be formed into a sphere, and it had good compressive strength.

The porous ceramsite’s mass loss rate during sintering also indicated the reaction characteristics of the primary substances during the sintering reaction. The mass loss rate decreased within 10–20 min of sintering. This indicated that AlN oxidation increased the sample weight [26]. However, the sample’s total mass still showed a mass loss, meaning that the release of volatile substances, such as water or salt, in the raw materials dominated. For example, the mass loss rate reached the maximum at 40 min of sintering. This result indicates that the release of volatile substances in the ceramsite was stronger than the AlN oxidation reaction.

### 3.3. Effect of Particle Size on Compressive Strength of Porous Ceramsite

The porous ceramsite’s particle size was an important factor that affected the ceramsite molding. The ceramsite’s compressive strength in the particle size range of 0.5–2 cm showed an upward trend (Table 4). Additionally, the mass loss rate decreased in the 1–2 cm section. The results indicate that the AlN oxidation reaction that produced N_2_ inside the ceramsite dominated and formed silicon–aluminum compounds [27] as the particle size increased. This was beneficial to increase the strength. For example, MgAl2O4 will be formed when synthetic porous glass ceramics are used by SAD [20].

### 3.4. Effect of Si/Al Ratio on Ceramsite’s Compressive Strength

The raw material composition’s characteristics were key factors in the formation of porous ceramsite [28]. Ceramsite could not form when the Si/Al ratio was less than two. Therefore, the compressive strength could not be measured. It may be that the Si/Al ratio was too low to form a molten state. The ceramsite’s compressive strength decreased with an increase in the Si/Al ratio when the Si/Al ratio was 2–3 (Table 5). It may have been that the Si/Al ratio was too high, and the brittleness of the ceramsite increased. This resulted in a decrease in its overall compressive strength [29]. Other researchers have also found that the large amount of SiO_2_ contained in the sample formed a “liquid film” [30,31,32], which hindered the N_2_ release produced by AlN oxidation. This resulted in more large bubbles inside the ceramsite, which affected the porous ceramsite’s compressive strength. In other words, the compressive strength of the ceramsite was affected by two reaction processes: (1) the more molten “liquid phase” wrapped the bubbles to form a porous structure, resulting in a decrease in compressive strength; and (2) the less molten “liquid phase” had difficultly in wrapping the bubbles, resulting in an increase in compressive strength.

The results regarding the effects of different Si/Al ratios on the compressive strength of ceramsite are shown in Table 5. When the Si/Al ratio was 1–3, as the Si/Al ratio increased, and the ceramsite’s mass loss rate showed an overall increasing trend, reaching the maximum when the Si/Al ratio was 3. Increasing the Si/Al ratio was beneficial in reducing the temperature of the molten state of the ceramsite. This allowed the release of more volatile substances such as water or salt. Therefore, the mass loss rate of the sample increased. An aluminosilicate melt “solution” environment during the SAD thermal reaction was proposed by Hanlin Shen [32], in which the SAD system would easily melt.

### 3.5. Effect of Multi-Factor Interaction on Ceramsite’s Compressive Strength

**Figure 3:**
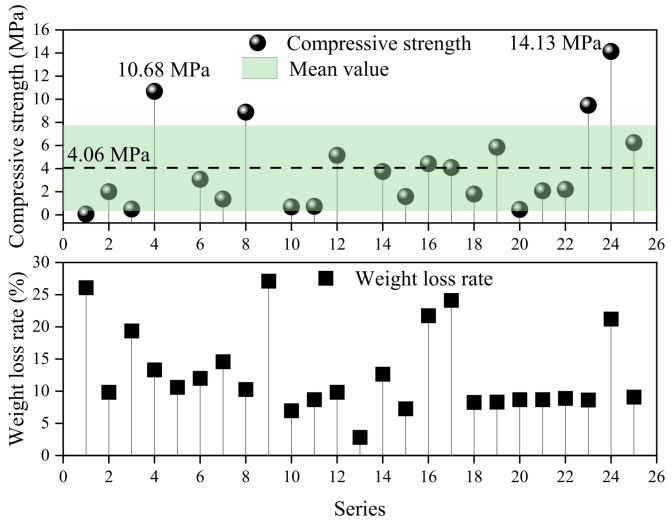
Orthogonal test result summary.

An orthogonal test was conducted to study the influence of the Si/Al ratio, sintering temperature, particle size, and sintering time on the compressive strength of porous ceramsite. Figure 3 shows that, under the experimental conditions, the average compressive strength of porous ceramsite was 4.06 ± 3.71 MPa. The compressive strength of the porous ceramsite obtained by SAD was higher than that of the ceramsite prepared from other types of solid waste. For example, the compressive strength of Yellow River sediment porous ceramsite was 1.1 MPa [33]. The maximum compressive strength of porous ceramsite was 14.13 MPa in this experiment. The sintering conditions were as follows: a sintering temperature of 1270 °C, a particle size of 2 cm, a sintering time of 30 min, and a Si/Al ratio of 1.5. Therefore, porous ceramsite produced with SAD could be achieved using general solid waste, and a corresponding pilot test should be conducted according to the experimental conditions.

SPSS was used to analyze the correlations between multiple factors and the compressive strengths. Table 6 shows that the Pearson’s correlation coefficients from the ceramsite compressive strength to the sintering temperature, particle size, sintering time, and silicon–aluminum ratio were 0.277, 0.611, 0.113, and 0.476, respectively. Therefore, it can be concluded that these factors affect the compressive strength of ceramsite in the following order according to their degree of contribution: particle size > Si/Al > sintering temperature > sintering time.

**Figure 4:**
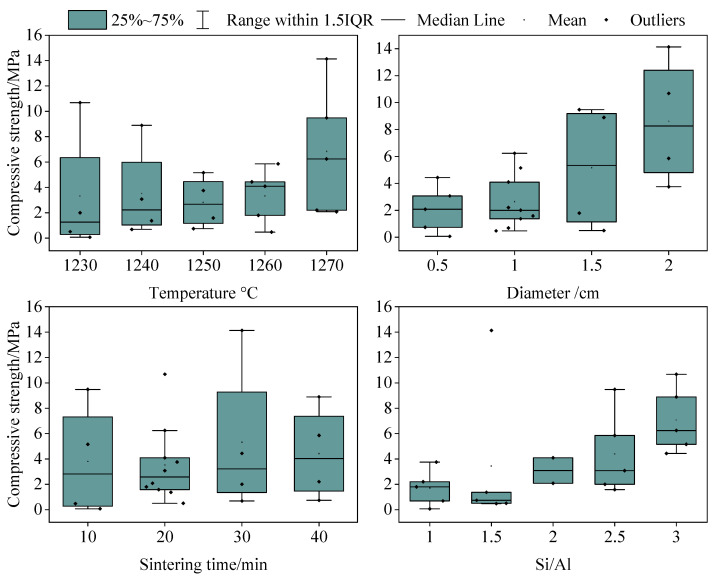
Relationship between multiples factors and the compressive strength in the orthogonal experiment.

The interactive effects of different factors on the compressive strength were analyzed based on the orthogonal experiments’ results. The results showed that, with an increase in the particle size and Si/Al ratio, the compressive strength value and its difference under the action of multiple factors increased. Figure 4 shows that, when the particle size was the smallest (0.5 cm), the compressive strength was the smallest and the deviation was the smallest (2.08 ± 1.76 MPa). When the particle size was the largest (2 cm), the compressive strength was the largest and the deviation was the largest (8.60 ± 4.06 MPa). The Si/Al ratio of the raw material exhibited a similar pattern to the particle size. When the Si/Al ratio was the smallest (2), the compressive strength was the smallest and the deviation was the smallest (3.09 ± 1.42 MPa). When the Si/Al ratio was the largest (3), the compressive strength was the largest and the deviation was large (7.08 ± 2.35 MPa). Therefore, an increase in the particle size and Si/Al ratio will promote the interactive effects of multiple factors on the compressive strength of ceramsite. A metakaolin-based geopolymer’s compressive strength was also found to systematically increase with increasing molar Si/Al ratios [34]. The average compressive strength in the literature [35] ranged from 9.69 MPa to 17.16 MPa, which was higher than that found in this study. This may have been related to factors such as the material properties. In the literature [36], the waste type, such as sewage sludge, and the mineral fraction of municipal waste were very important, and they determined the composition of the ceramsite, which further affected its compressive strength.

However, although the influence of multiple factors on the compressive strength varies greatly when adjusting the sintering temperature and time, the influence of the temperature and sintering time on this difference did not show a significant correlation (Figure 3). When the sintering temperature was 1250 °C, the standard deviation of the compressive strength was the smallest (2.81 ± 2.01 MPa). This meant that the differences in the compressive strength of different ceramsite particles at this temperature were the smallest, but the differences in the compressive strength did not increase with the increasing temperature. Similarly, the standard deviation of the compressive strength was the smallest (3.52 ± 3.02 MPa) when the sintering time was 20 min. However, the sintering time with the largest compressive strength value and its deviation (5.31 ± 5.26 MPa) was 30 min instead of 40 min.

Therefore, the particle size and Si/Al ratio had a greater influence on the compressive strength, which is similar to the results of the correlation analysis above. This indicates that more attention should be paid to the changes in the particle size and the Si/Al ratio during porous ceramsite preparation using SAD.

## 4. Conclusions

Porous ceramsite was prepared using SAD in this study. A combination of single-factor experiments and orthogonal experiments was conducted to explore the influence of the sintering conditions on the compressive strength. In this study, the influence of a single factor on the compressive strength of ceramsite and the synergistic relationships between different factors were investigated. The following conclusions were drawn.

1. The level of each factor showed an overall positive correlation with the ceramsite’s compressive strength within a certain range of variation. Based on this, the sintering conditions for the highest porous ceramsite compressive strength of 14.13 MPa are proposed as follows: sintering temperature, 1270 °C; particle size, 2 cm; sintering time, 30 min; and Si/Al ratio, 1.5.
2. The contribution of each factor to the compressive strength of porous ceramsite was in the following order: particle size > Si/Al ratio > sintering temperature > sintering time. The average compressive strength of porous ceramsite obtained under the action of multiple factors was 4.06 ± 3.71 MPa.
3. There was a synergistic effect among the factors. An increase in the particle size and Si/Al ratio will promote the interaction of multiple factors regarding the compressive strength of ceramsite. With increases in the particle size and the Si/Al ratio, the compressive strength and its difference in the porous ceramsite increased. More attention should be paid to the influence of the particle size and Si/Al ratio in the actual sintering of porous ceramsite.
4. In addition to the synergistic effects of multiple factors, there was also a relationship between the compressive strength and the ceramsite’s porous state during the sintering process of SAD-based porous ceramsite. Pilot or industrial tests should be conducted in the future based on these experiments and the intended use of porous ceramsite.

## Figures and Tables

**Table 1:** Summary of orthogonal tests.

Series	Temperature/°C	Particle Size/cm	Sintering Time/min	Si/Al
1	1230	0.5	10	1
2	1230	1	30	2.5
3	1230	1.5	20	1.5
4	1230	2	20	3
5	1230	1	40	2
6	1240	0.5	20	2.5
7	1240	1	20	1.5
8	1240	1.5	40	3
9	1240	2	10	2
10	1240	1	30	1
11	1250	0.5	40	1.5
12	1250	1	10	3
13	1250	1.5	30	2
14	1250	2	20	1
15	1250	1	20	2.5
16	1260	0.5	30	3
17	1260	1	20	2
18	1260	1.5	20	1
19	1260	2	40	2.5
20	1260	1	10	1.5
21	1270	0.5	20	2
22	1270	1	40	1
23	1270	1.5	10	2.5
24	1270	2	30	1.5
25	1270	1	20	3

**Table 2:** Effects of different sintering temperatures on ceramsite compressive strength.

Si/Al	Particle Size (cm)	Sintering Time (min)	Sintering Temperature (°C)	Mass Loss Rate (%)	Compressive Strength (MPa)
2	2	20	1230	26.44	4.08
			1240	29.29	4.99
			1250	29.97	3.14
			1260	27.50	13.14
			1270	27.87	17.07

**Table 3:** Effects of different sintering times on the ceramic pellets’ strength.

Si/Al	Particle Size (cm)	Sintering Temperature (°C)	Sintering Time (min)	Mass Loss Rate (%)	Compressive Strength (MPa)
2	2	1260	10	28.48	6.17
			20	27.50	13.14
			30	28.64	—
			40	31.48	—

**Table 4:** Effects of different particle sizes on the ceramic granules’ strength.

Si/Al	Sintering Time (min)	Sintering Temperature (°C)	Particle Size (cm)	Mass Loss Rate (%)	Compressive Strength (MPa)
2	20	1260	0.5	27.51	1.39
			1.0	28.64	2.76
			1.5	27.11	5.27
			2.0	27.50	13.14

**Table 5:** Effects of different raw material ratios on ceramic pellets’ strength.

Sintering Temperature (°C)	Particle Size (cm)	Sintering Time (min)	Si/Al	Mass Loss Rate (%)	Compressive Strength (MPa)
1260	2	20	1	26.17	—
			1.5	28.77	—
			2	27.50	13.14
			2.5	30.17	6.61
			3	32.34	3.73

**Table 6:** Summary of the correlations between the sintering conditions and compressive strength.

Condition	Correlation	Temperature (°C)	Particle Size (cm)	Sintering Time (min)	Si/Al	Compressive Strength (Mpa)
Temperature/°C	Pearson correlation	1	0.00	0.00	0.00	0.28
	Sig.		1.00	1.00	1.00	0.211
Particle size /cm	Pearson correlation	0.00	1	0.00	0.00	0.61 **
	Sig.	1.00		1.00	1.00	0.003
Sintering time /min	Pearson correlation	0.00	0.00	1	0.00	0.11
	Sig.	1.00	1.00		1.00	0.618
Si/Al	Pearson correlation	0.00	0.00	0.00	1	0.48 *
	Sig.	1.00	1.00	1.00		0.025

**, the correlation was significant at the 0.01 level; *, the correlation was significant at the 0.05 level.

## Data Availability

Data are provided in the article.

## Abbreviations

PAD	primary aluminum dross
SAD	secondary aluminum dross
AlN	aluminum nitride
Si/Al	silicon–aluminum ratio
